# Comprehensive bioinformatics analysis and experimental verification identify mitochondrial gene Dgat2 as a novel therapeutic biomarker for myocardial ischemia-reperfusion

**DOI:** 10.3389/fendo.2025.1539646

**Published:** 2025-05-29

**Authors:** Hong Li, Yixin Zhou, Wenya Xue, Pan Yin, Luyu Liu, Shaobo Wu, Yahao Zhao, Qi An, Yang Sun

**Affiliations:** ^1^ Department of General Medicine, Xijing Hospital, The Fourth Military Medical University, Xi’an, China; ^2^ Department of Geriatrics, Xijing Hospital, The Fourth Military Medical University, Xi’an, China; ^3^ Department of Medicine, Xi’an Jiaotong University, Xi’an, Shaanxi, China

**Keywords:** myocardial ischemia/reperfusion injury, mitochondria, Dgat2, bioinformatics analysis, oxidative stress

## Abstract

**Background:**

Ischemic cardiomyopathy is a severe disease marked by high morbidity and mortality, often exacerbated by myocardial ischemia/reperfusion injury (MI/RI). Mitochondrial metabolism plays a critical role in MI/RI progression. This study aimed to identify potential new targets and biomarkers for mitochondria-related genes in MI/RI.

**Methods:**

MI/R microarray data (GSE160516) from the GEO database and a mitochondrial geneset were analyzed. Limma identified differentially expressed genes (DEGs), followed by GSEA, GO, and KEGG pathway enrichment. Mitochondria-related DEGs (MitoDEGs) were pinpointed. Protein-Protein Interaction (PPI) networks and machine learning identified key MitoDEGs. Regulatory networks were constructed using transcription factor (TF) predictions. Immune cell infiltration was assessed with ImmuCelAl, and correlations between MitoDEGs and immune cell levels were examined. Mouse myocardial ischemia-reperfusion models were established to validate pivotal MitoDEGs.

**Results:**

MitoDEGs were enriched in bio-oxidation, immune-inflammation, and oxidative stress pathways. Machine learning identified two hub genes: Dgat2 and Cybb. Dgat2 was significantly elevated in ischemia-reperfusion mouse models, confirmed by RT-PCR and Western blot. Functional enrichment indicated that Dgat2 may be involved in biological oxidation and lipid metabolism. TF prediction suggested PPARG as a regulator of Dgat2 expression. Immune infiltration analysis revealed significant correlations between Dgat2 and immune cells, including CD4_T_cells and NK cells, suggesting a role for immunity in MI/RI.

**Conclusions:**

We found that Dgat2 could be exploited as a novel mitochondria-related gene target and biomarker in myocardial ischemia-reperfusion injury, which is of great clinical significance.

## 1 Introduction

Ischemic heart disease remains one of the leading causes of death globally (1), with the World Health Organization (WHO) estimating that more than 9 million people died from the disease in 2016. Myocardial ischemia/reperfusion injury (MI/RI) is one of the most common fatal complications (2), resulting from myocardial cell injury and inflammation due to blood and oxygen reperfusion following myocardial ischemia (3). Myocardial infarction, as a typical manifestation of ischemic heart disease, begins with reversible ischemic injury and progresses to irreversible injury, ultimately leading to fibrous scar formation (4). Although pharmacological and interventional therapies have significantly improved survival in cardiovascular disease (5), there is a lack of specific therapies for MI/RI (6). Therefore, elucidating the molecular mechanisms of MI/RI, identifying reliable biomarkers, and exploring potential therapeutic targets are critical.

Mitochondria play a central role in myocardial ischemia/reperfusion injury (7–11). As an energy-intensive organ, the myocardium is rich in mitochondria (12), which are not only responsible for ATP production, but also involved in the regulation of reactive oxygen species (ROS). Studies have shown that the overproduction of ROS during reperfusion especially the activation of the NOX family (13) and mitochondrial respiratory chain is a key pathological mechanism of MI/RI (14). Physiologically, ROS regulates processes such as cardiac development and cardiomyocyte maturation (15, 16); However, under pathological conditions, ROS imbalance triggers oxidative stress, leading to oxidative damage to DNA, proteins, and lipids, and activating mitochondrial permeability transition pore (MPTP) (17). The opening of MPTP prompts lysosomes to engulf mitochondria, leading to abnormal mitochondrial morphology and loss of kinetic proteins (18), ultimately leading to cardiomyocyte energy metabolism disorders and death (19–21). Notably, mitochondrial dynamic imbalance in ischemic heart disease has been shown to be closely related to cardiac insufficiency (22), suggesting that mitochondria play multiple regulatory roles in maintaining cardiac function.

In this study, we aimed to explore potential mitochondrial gene targets and biomarkers related to mitochondria in myocardial ischemia/reperfusion through bioinformatics analysis and molecular experiments, in order to reveal the association mechanism between the two at the molecular level.

## 2 Materials and methods

### 2.1 Data processing

The MI/R dataset was obtained from the public database NCBI GEO (http://www.ncbi.nlm.nih.gov/geo) (23) using “myocardial ischemia-reperfusion” as the search term. We further filtered them based on the information of sequencing type (transcriptomics), animal species (mouse), sample source (heart) and modeling time. Finally, GSE160516 was obtained. All mice of GSE160516 were euthanized with an overdose of pentobarbital at hours 6, 24, and 72 of ischemia-reperfusion (I/R) treatment. Total RNA was isolated from cardiac organs (n=4 per group) at each time point using TRIzol (Invitrogen) according to the manufacturer’s instructions. Gene expression profiling was performed using Affymetrix GeneChip mouse Genome 430 2.0 arrays (Affymetrix, Inc.) on the GeneChip Mouse Genome 430 2.0 Array, over 45,000 probe sets were analyzed for expression levels of over 39,000 transcripts and variants in over 34,000 well-characterized mouse genes. Mice euthanized 24 h after sham-operated treatment and I/R treatment (n=4 per group) were selected for this study.

We also obtained a set of mitochondria-related genes from 2031 mitochondria-related papers (https://pubmed.ncbi.nlm.nih.gov/36915111/) for subsequent analysis together with MI/RI-related genes.

### 2.2 Identification of differentially expressed genes

DEGs from the dataset were obtained with R package “limma” as implemented by Sangerbox (http://www.sangerbox.com/tool) (24), and all identified DEGs met *p*<0.05 and |log2 (Fold-change)|≥1.5. Meanwhile, we used Sangerbox to plot Volcano Plot and Heatmap respectively.

### 2.3 Functional enrichment analysis

Sangerbox (http://www.sangerbox.com/tool) was used for Gene Set Enrichment Analysis (GSEA), Gene Ontology (GO) and Kyoto Encyclopedia of Genes and Genomes (KEGG) enrichment analysis. Gene Set Enrichment Analysis (GSEA) is a computational method that determines whether an *a priori* defined set of genes shows statistically significant, concordant differences between two biological states (e.g. phenotypes). Gene Ontology (GO) analysis is a common technique utilized for conducting large-scale functional enrichment studies that encompass biological processes, molecular functions, and cellular components (25). Kyoto Encyclopedia of Genes and Genomes (KEGG) is a popular database for storing information pertaining to genomes, biological pathways, diseases, and pharmaceuticals (26). Adjusted P-value < 0.05 was considered significant.

### 2.4 STRING database analysis

STRING (https://string-db.org) is an online database that is commonly used for creating Protein-Protein Interaction (PPI) networks and scoring each interaction between target proteins (27). We performed a PPI network analysis of Dgat2 to investigate its interaction with STRING at a confidence level of greater than 0.9.

### 2.5 GeneMANIA database analysis

GeneMANIA (http://www.genemania.org) is a user-friendly website that provides information on protein and genetic interactions, pathways, co-expression, co-localization, and protein domain similarity for inputted genes (28). In this investigation, 20 direct neighbor genes of Dgat2 in the PPI network were identified using GeneMANIA.

### 2.6 Machine learning

Machine learning algorithms are used to screen core genes for diagnostic purposes. We filtered down the candidate biomarkers using the Random Forest (RF) technique, which incorporates many trees for improved accuracy via ensemble learning (29). The genes with MeanDecreaseGini > 2 in the RF model were designated as key genes.

### 2.7 Immune infiltration analysis

The website ImmuCellAI (https://github.com/lydiaMyr/ImmuCellAI) and ImmuCellAI-mouse (https://guolab.wchscu.cn/ImmuCellAI-mouse//#!/) is utilized for evaluating the percentage of immune cells present in cells or tissues, which simulated the flow cytometry process to predict cell type abundance by hierarchical strategy. 24 immune cells are included: B_cell, Dendritic_cells, Granulocytes, Macrophage, Monocytes, NK, T_cell, CD4_T_cell, CD8_T_cell, NKT, Tgd, Follicular_B, Germinal_center_B, Marginal_Zone_B, Memory_B, Plasma_cell, cDC1, cDC2, MoDC, pDC, Basophil, Eosinophil, mast_cell, Neutrophils, M1_macrophage, M2_macrophage, CD4_Tm, Naive_CD4_T, T_helper_cell. The bar graphs show the proportion of each type of immune cell in various samples, and the “corrplot” R package is used to generate a heat map of the correlation between 24 immune cells. Spearman’s method was used to study the relationship between MitoDEGs and Immune cells infiltration level.

### 2.8 Identification of transcription factors interacting with key genes and prediction of interaction sites

We used three different databases, GTRD, ChEA3, and hTFtarget, for initial transcription factor prediction.

hIP-X Enrichment Analysis 3 (ChEA3, https://amp.pharm.mssm.edu/ChEA3) is a transcription factor enrichment analysis tool that ranks TFs associated with user-submitted gene sets. The ChEA3 background database contains a collection of gene set libraries generated from multiple sources, including from RNA-seq studies, TF-gene co-expression, TF-target associations from ChIP-seq experiments, and TF-gene co-occurrence calculated from multiple submitted gene lists.

The GTRD database (http://gtrd.biouml.org/) collects transcription factor-associated chip_seq data from public databases such as SRA, GEO, ENCODE, etc., performs peak calling analysis using a standard process, and predicts transcription factor binding sites TFBS based on available transcription factor motif data.

The hTFtarget (http://bioinfo.life.hust.edu.cn/hTFtarget) database integrates large-scale human TF target resources and epigenetic modification information to predict precise TF-target regulatory relationships.

UCSC (http://genome.ucsc.edu/) contains a large amount of genomic data, including gene annotation information (ENCODE), genome-to-genome comparison information, repetitive sequences, homologous sequences, reference sequences (mRNA, EST), phenotypes, expression profiles, regulatory information, conservatism, variants, repetitive regions, and a series of other information, which were used in this study to prediction of transcription factors and their binding DNA sequences.

We performed transcription factor-promoter binding site analysis using JASPAR (https://jaspar.elixir.no) and the online transcriptional regulation analysis website (https://jingege2.shinyapps.io/TF_targets_predict/) for hub transcription factor (TF) DNA binding sequence prediction.

### 2.9 MI/RI surgery

Mice were anesthetized with 1.5% isoflurane and fixed in the supine position. The left chest wall area was shaved and the skin was cut to expose the chest wall. The heart was gently extruded out through the 4th intercostal space by a hemostatic forcep. The left anterior descending coronary artery was ligated using a 6–0 suture. The position of the ligation site was kept consistent among each mouse. The slip knot was gently loosened to reperfused the myocardium after 30 min of the ischemia. The heart was softly pushed back to the chest and the chest wall was sutured with a 4–0 suture. Cardiac function, infarct size, the expression of cleaved caspase-3 and lactic dehydrogenase were evaluated 24 h after the reperfusion.

### 2.10 Echocardiographic assessment

Mice were anesthetized with 1.5% isoflurane 24 h after the reperfusion, and echocardiography was performed using the VEVO 770 high-resolution imaging system (Visual Sonics, Canada). A two-dimensional M-mode image was used to evaluate the left ventricular function. On the parasternal LV long-axis view, the left ventricular end-systolic diameter (LVESD) and left ventricular end-diastolic diameter (LVEDD) were measured. The ejection fraction (EF) and fractional shortening (FS) parameters were calculated using the computerized algorithms. The results were averaged from the five consecutive cardiac cycles measured from the M-mode images.

### 2.11 Measurements of serum LDH activity

After cardiac reperfusion for 24 h, blood samples were collected and the serum was separated by 1,000 rpm centrifugation for 10 min. LDH activity in serum were detected by using LDH Assay kit (C0016, Beyotime), according to the manufacturers’ instructions.

### 2.12 Measurement of myocardial infarct size

The infarct size was measured by the Evans blue/2,3,5-triphenyl tetrazolium chloride (TTC) double staining. In brief, 10μL of 1% Evens blue dye were injected into the aorta root after the left coronary artery was fully ligated. The heart was quickly excised and frozen after the Evens blue dye was distributed into the whole heart. The heart was transversally cut into five successive 1 mm-thick slices. The slices were incubated in 1% TTC staining dye at 37°C for 10 min. The area at risk (AAR) regions were the Evans blue-unstained regions, and the infarct areas (INF) were the pale white regions. The INF and AAR were measured by a blinded observer using Image-Pro 6.0 Plus software. The 3rd slice of the five successive slices was chosen to measure the INF/AAR ratios.

### 2.13 Quantitative real-time PCR

The total RNA of frozen tissues was isolated using the RNAiso Plus (#9109, Takara) and extracted using the RNeasy Mini Kit (#74106, Qiagen) in accordance with the manufacturer’s protocol. The reverse transcription of RNA was performed using the PrimeScriptTM RT Reagent Kit with gDNA Eraser (#RR047A, Takara). The primers were designed and synthesized by the Tsingke Biotechnology, Beijing, China. RT–PCR was performed using the TB Green Premix Ex Taq II kit (#RR820A, Takara) and the CFX Real-Time PCR System (Bio-Rad, US). The thermocycle conditions were set according to the manufacturer’s protocol. The pre-denaturation was performed at 95°C for 30 seconds, the amplification was performed at 95°C for 5 seconds and 60°C for 30 seconds for 40 cycles. The mRNA expression was calculated using the formula 2(-△Ct). The relative change of mRNA expression was calculated using the formula 2(-△△Ct) by normalizing to the control group.

### 2.14 Western blotting

The frozen ventricular tissues were homogenized in the Cell 97 Lysis Buffer (#9803, CST) supplemented by 1% phosphatase/proteinase inhibitor cocktail (#78438, ThermoFisher). The 20 to 50μg proteins were mixed with reducing sample buffer and desaturated by boil. Proteins were separated by SDS-PAGE gel, and transferred onto a methanol-treated 0.22μm PVDF membrane. The membrane was blocked with 5% nonfat milk dissolved in Tris-buffered saline-Tween 20 solution for 1 hour at room temperature. The members were incubated with the primary antibodies for 24 h at 4°C. After incubating with the secondary HRP-conjugated antibodies (#ab205719 and #ab205718, abcam) for 1 hour at room temperature, the protein bands were visualized and analyzed by chemiluminescent (ECL) detection system (Image Lab, Bio-Rad, US).

The primary antibodies include: vinculin (sc-73614, santa cruz), cleaved caspase-3 (sc-56053, santa cruz), Dgat2(17100-1-AP, proteintech), Cybb(K002889P, Solarbio).

### 2.15 Immunohistochemistry

Hearts were fixed, paraffin-embedded, and sectioned for immunohistochemistry. After dewaxing and rehydration, citrate buffer was used to restore the antigens, and the sections were incubated with Endogenous Peroxidase Blocking Buffer (Beyotime, P0100B). Next, peroxidase activity was inactivated, and tissues were blocked using QuickBlock™ Blocking Buffer and incubated with Dgat2 (proteintech, 17100-1-AP, 1:200 dilution)overnight at 4°C. To visualize the target protein, for Dgat2, a DAB solution was applied, followed by hematoxylin staining. The sections were dehydrated and sealed.

## 3 Results

### 3.1 DEGs and functional enrichment analysis in MI/RI

**Figure 1:**
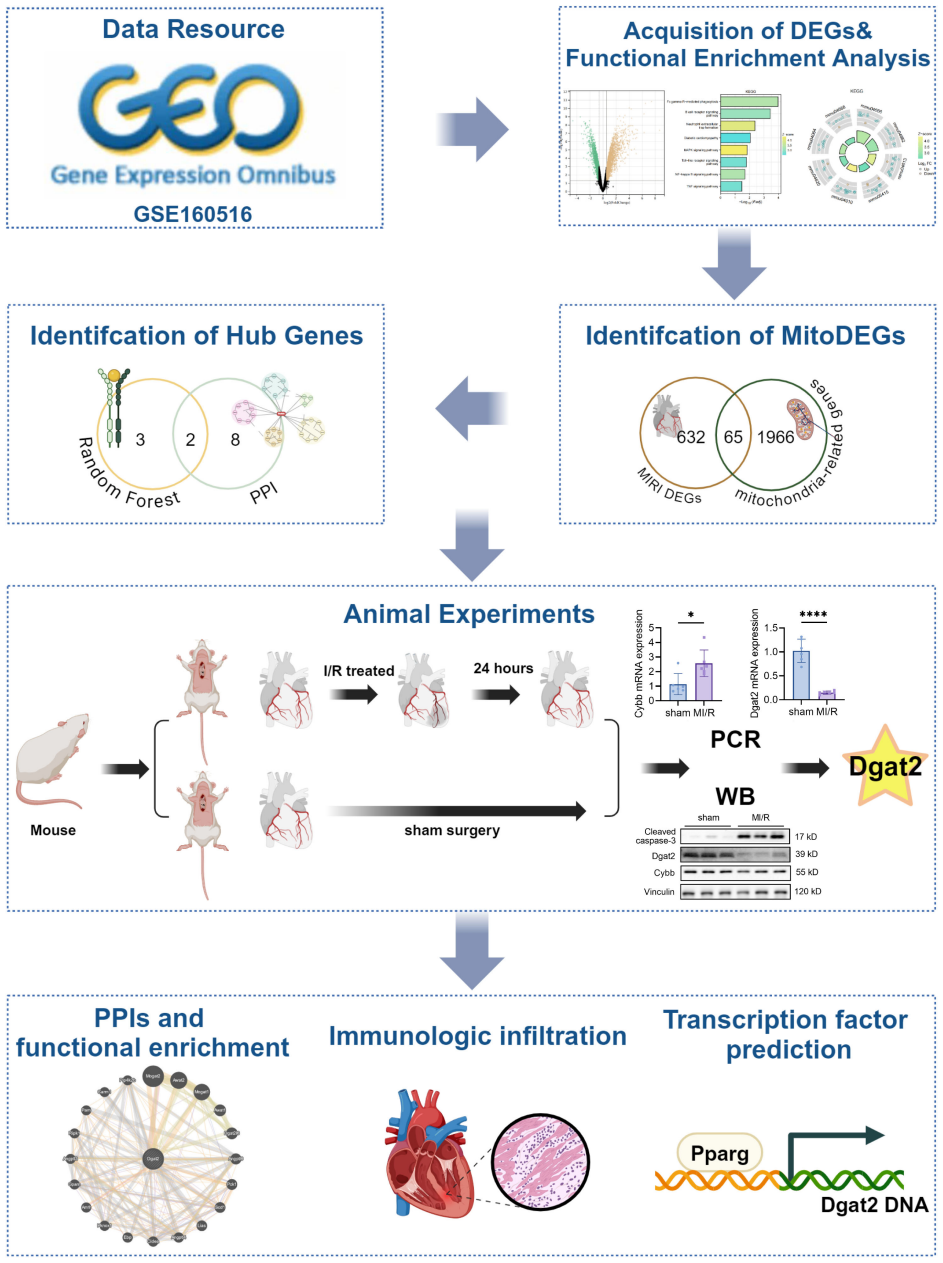
Flowchart of analytical steps in this study.

**Figure 2:**
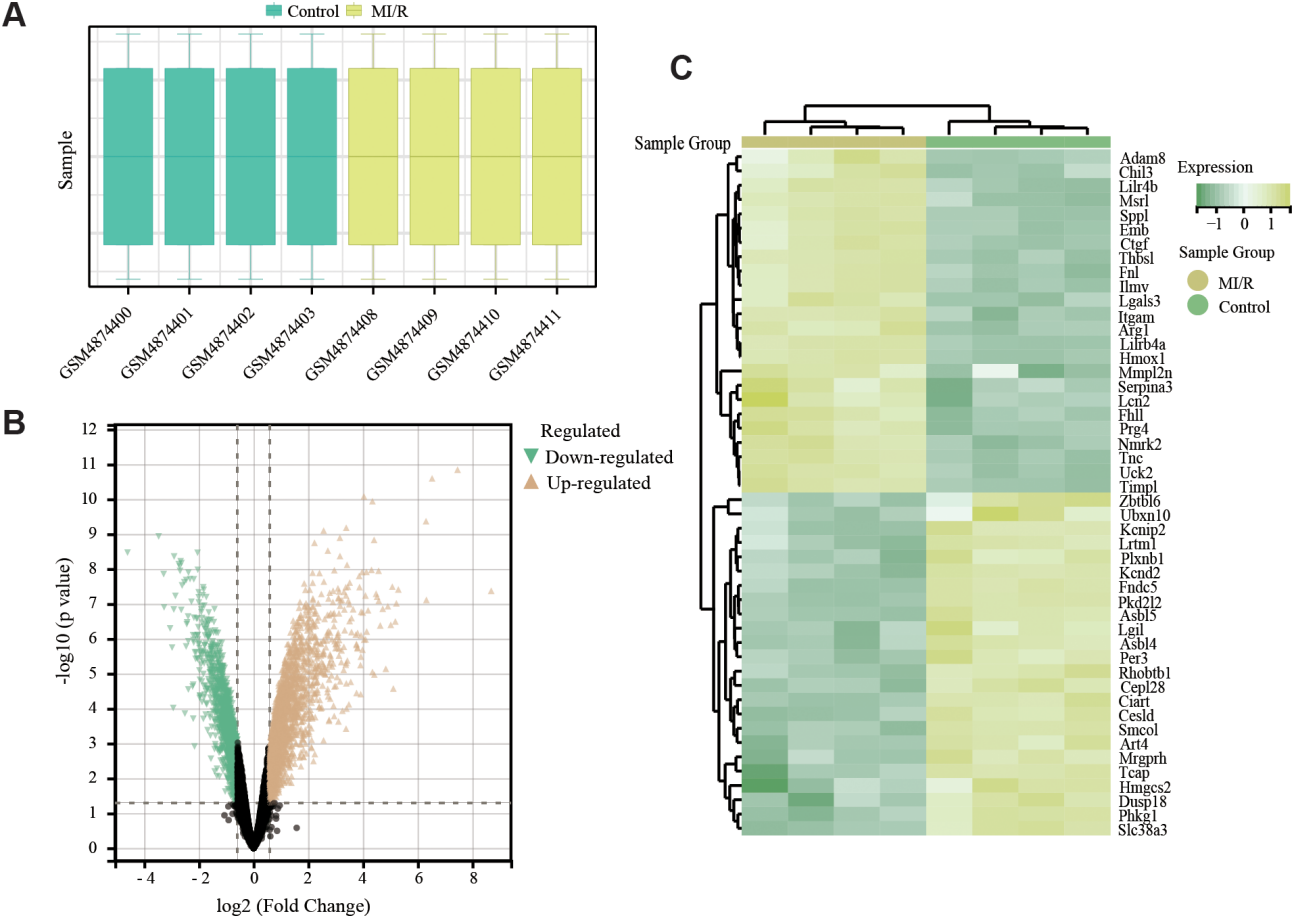
Sample characteristics and identified DEGs in MI/RI. **(A)** Individual characteristics of the GSE dataset. **(B)** Volcano plot of DEGs in GSE160516. Orange triangles and green triangles represent significantly upregulated and downregulated genes, respectively. Black dots: non-significant. Dashed lines indicate thresholds. **(C)** Clustered heatmap of DEGs in GSE160516. Color scale: relative expression (yellow=high, green=low). Rows: DEGs; columns: samples (yellow=MI/R, green=control).

The whole data screening strategy flowchart is depicted in **Figure 1**. We obtained the GEO dataset GSE160516 related to MI/RI for analysis. We used data from 4 sham-operated mice (GSM4874400, GSM4874401, GSM4874402, GSM4874403) and 4 mice (GSM4874411, GSM4874410, GSM4874408, GSM4874409) from this dataset at 24 h after I/R treatment (**Figure 2A**). Analysis of variance revealed that with logFC set to 1.5, there were 697 DEGs in the MI/RI samples, comprising 530 genes up-regulated and 167 genes down-regulated as compared to the normal samples. The DEGs were presented as volcano and heat maps (**Figures 2B, C**).

### 3.2 GO and KEGG pathway analysis and GSEA of DEGs

**Figure 3:**
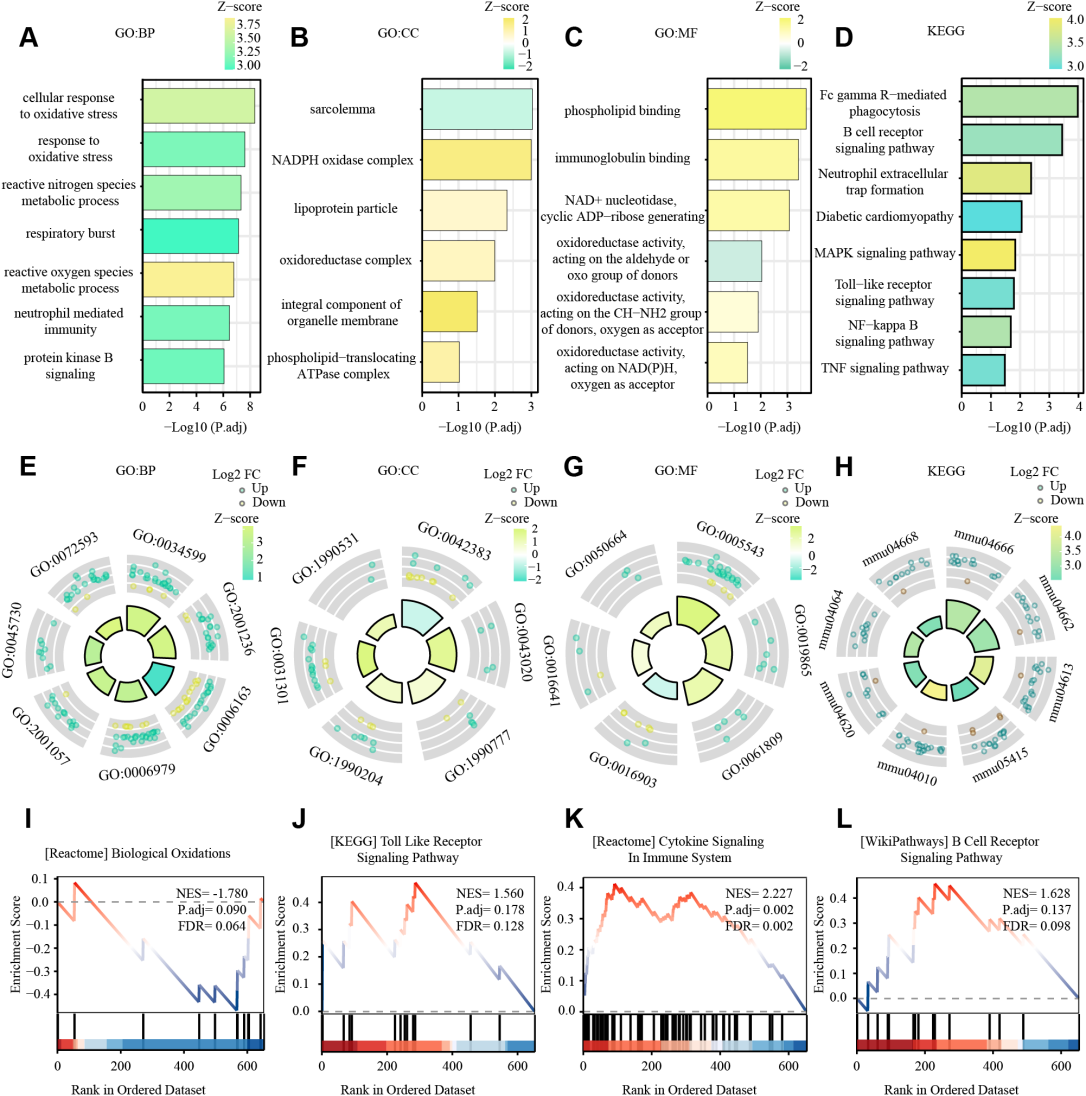
GO and KEGG enrichment analyses of DEGs from GSE160516, and results of GSEA analysis. **(A, E)** The enriched GO: BP (biological process) terms of DEGs in GSE160516. In **(A)** the terms significantly enriched are sorted by Z-score (color bar). The x-axis represents statistical significance (-Log10 adjusted p-value), and the y-axis shows the GO terms. Key pathways related to oxidative stress and immune response are highlighted. In **(E)** the GO terms are color-coded based on regulation direction (upregulated: green; downregulated: yellow), with the size/height of the bars representing the Z-score. Several terms and their GO identifiers are highlighted. **(B, F)** The enriched GO: CC (cellular component) terms of DEGs in GSE160516. **(C, G)** The enriched GO: MF (molecular function) terms of DEGs in GSE160516. **(D, H)** KEGG pathway enrichment results in GSE160516. **(I-L)** 4 GSEA datasets(Biological Oxidations, Toll Like Receptor Signaling Pathway, Cytokine Signaling Immune System, B Cell Receptor, Signaling Pathway) with significant correlation. The curves in the figure display the distribution of genes in the ranked dataset. The x-axis represents the gene ranking in the ordered dataset, and the y-axis represents the enrichment score. NES (Normalized Enrichment Score), P.adj, and FDR (False Discovery Rate) are indicated in the figure.

The DEGs were then processed for functional enrichment using GO and KEGG pathway analysis (**Figures 3A–H**). The most enriched GO terms were classified into biological processes (BP), cellular components (CC), and molecular functions (MF), among which we found the more interesting ones to be respiratory and oxidative stress-related pathways, intracellular redox processes, cellular respiration, intracellular signaling processes, energy metabolism, inflammation-immunity, immunity, and other related pathways. Fc gamma R-mediated phagocytosis, B cell receptor signaling pathway, Neutrophil extracellular trap formation, Diabetic cardiomyopathy, MAPK signaling pathway, Toll-like receptor signaling pathway, NF-kappa B signaling pathway, and TNF signaling pathway were the KEGG pathways with the highest DEG enrichment.

GSEA revealed that DEGs in the dataset were involved in oxidative stress-related pathways (e.g., biological oxidation), extracellular matrix change-related pathways (e.g., Integrin Cell Surface Interactions, NABA Matrisome Associated, NABA Matrisome), and immune-related pathways (e.g., neutral granulocyte degranulation, innate immune system) (**Figures 3I–L**).

### 3.3 MitoDEGs in MI/R and hub MitoDEGs identification

**Figure 4:**
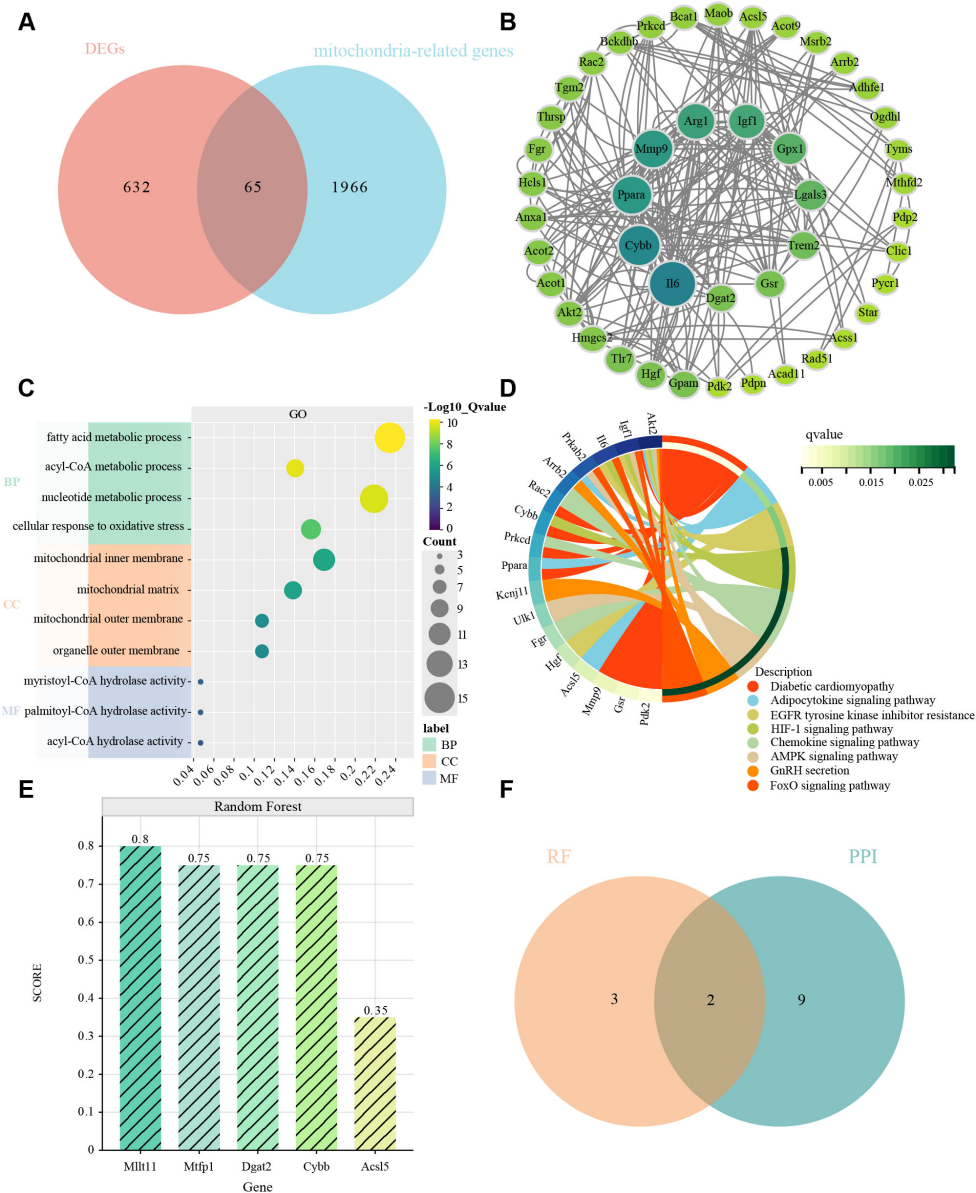
MitoDEGs in MI/RI; PPI network analysis, GO and KEGG enrichment analyses and hub MitoDEGs identification. **(A)** MitoDEGs that overlap between DEGs and mitochondria-related genes from 2031 mitochondria-related publications. **(B)** PPI network of MitoDEGs. The nodes represent genes, with the color intensity reflecting their significance (dark green indicates high significance, while light green indicates low significance). The edges represent interactions between the genes. **(C, D)** GO and KEGG pathway enrichment results in MitoDEGs. **(E)** Identification of five characterized genes in Random Forest pairs. The x-axis represents the genes, and the y-axis represents their scores in the Random Forest model. The darker the color of the bars, the higher the score. **(F)** Hub MitoDEGs that overlap between 10 key genes in PPI network and 5 major genes in Random Forest results.

We obtained a set of mitochondria-related genes from 2031 mitochondria-related papers, from which we selected genes overlapping with DEGs as MitoDEGs. 65 MitoDEGs were obtained in total (**Figure 4A**).

The 65 genes were uploaded to the STRING database to construct a protein-protein interaction network, and the key genes were found to be Il6, Cybb, Ppar, Mmp9, Arg1, Igf1, Gpx1, Lgals3, Trem2, Gsr, and Dgat2. We then used the CytoHubba application in Cytoscape to analyze using the “MNC” algorithm to identify key genes, with the color of the nodes indicating the strength of the correlation (**Figure 4B**).

For these 65 genes we then performed GO and KEGG analysis, which showed correlation with mitochondrial function, oxidative stress, signaling, fatty acid metabolism, nucleotide metabolism, and other processes (**Figures 4C, D**).

Screening by Random Forest pairs resulted in the identification of five characterized genes, including Dgat2, Cybb, Acsl5, Mtfp1, and Milt11(**Figure 4E**). Next, comparison of the Random Forest results with the PPI results revealed that these algorithms identified two overlapping genes (**Figure 4F**), i.e., Dgat2 and Cybb, which were used in the subsequent analyses.

### 3.4 General biological and echocardiography features of MI/R mouse

C57BL/6 J male mice (18–25 g, 6–8 weeks old) were obtained from the Laboratory Animal Center of the Fourth Military Medical University (Xi’an, China). The mice were separately maintained in an individually ventilated cage system with free access to the food and drinking water. They were raised in a 12 h:12 h light: dark cycle at 22–24°C. All animal experiments were carried out in agreement with the Guide for the Care and Use of Laboratory Animals published by the United States National Institutes of Health (NIH publication no. 86–23, revised 1996) and with the approval of the The Fourth Military Medical University Experimental Animal Research Committee.

**Figure 5:**
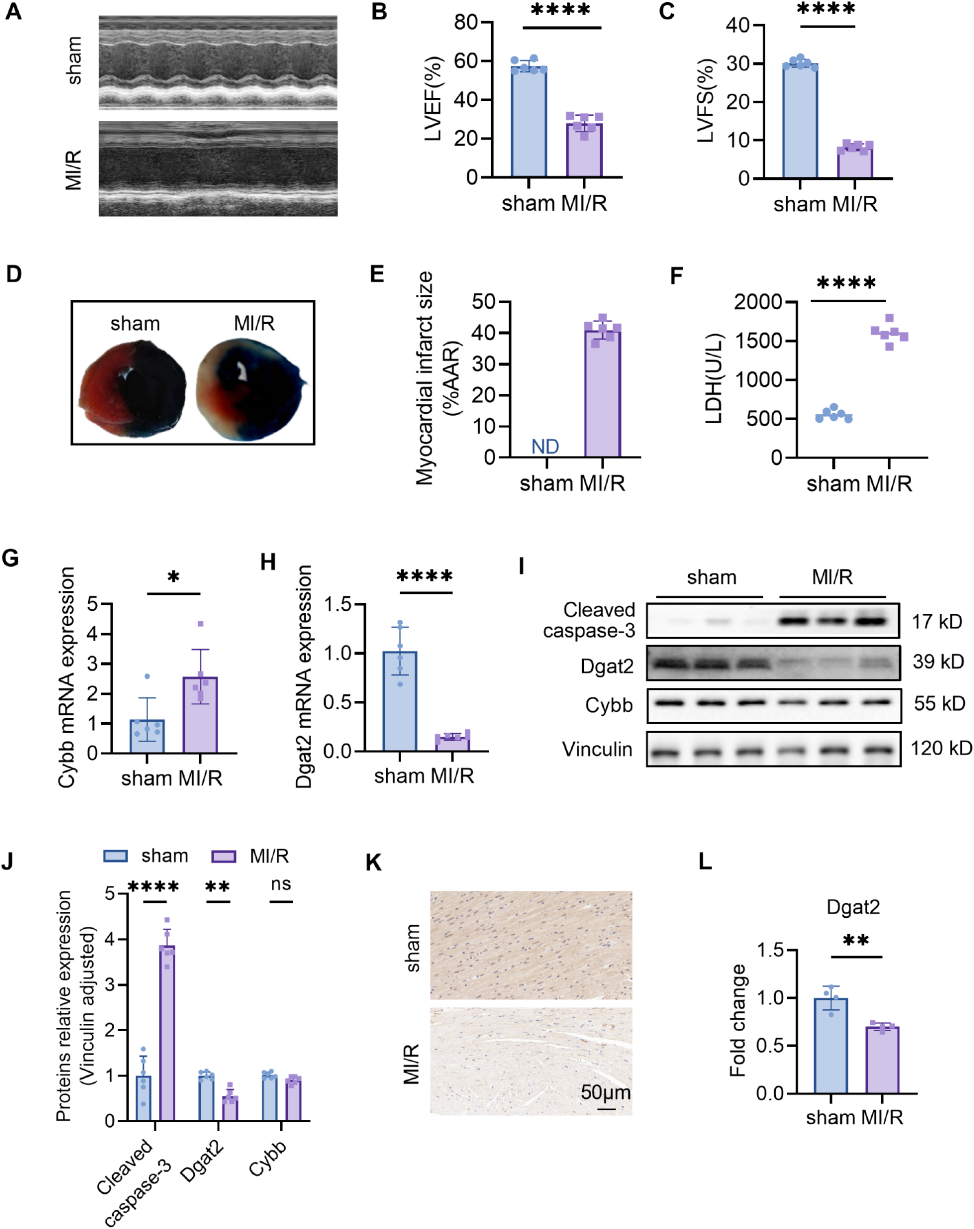
Confirmation of hub MitoDEGs expression and association with MI/R mouse. **(A)** Representative echocardiographic images were obtained 24 h after myocardial ischemia/reperfusion (MIR). **(B, C)** Left ventricular ejection fraction (LVEF) and fractional shortening (LVFS). **(D)** Representative images of myocardial 2,3,5-triphenyltetrazolium chloride (TTC)/Evans blue double staining. **(E)** The infarct size (INF) was calculated as a percentage of the myocardial area at risk (AAR). **(F)** The LDH levels in serum. **(G, H)** Cybb and Dgat2 relative mRNA expression. **(I, J)** Protein levels of Cybb, Dgat2 by western blotting, and quantitative analysis in heart tissue. **(K, L)** The immunohistochemistry staining of Dgat2 protein in heart tissue and quantitative analysis. Data are expressed as mean ± SE, n = 6, ns, no significance, *p < 0.05, **p < 0.01, ****p < 0.0001.

We conducted animal experiments. For the cardiac ischemia-reperfusion (I/R) model, surgeries were performed under isoflurane anesthesia and using a ventilator to acquire passive respiration. Cardiac I/R was induced by ligation of the left anterior descending artery (LAD) for 30 min followed by cardiac reperfusion for 24 h (acute I/R injury). Compared to the CON group, the MI/R group witnessed significantly lower EF% and FS% (P<0.05) but remarkably higher infarct size (P<0.05) and lactic dehydrogenase level, as well as cleaved caspase-3 (**Figures 5A–F, I**).

### 3.5 Experimental validations of hub MitoDEG expression in MI/R mouse

Ventricular expression of 2 hub MitoDEGs (Dgat2 and Cybb) was validated in mouse with qRT-PCR. The primer sequences used for qPCR are listed in **Table 1**. As compared to the Control group, Cybb had significantly increased expression in the MI/R group (P<0.05), while Dgat2 reversely exhibited remarkably decreased expression in the MI/R group (P<0.05) (**Figures 5G, H**). After that, we further validated the protein expressions of Cybb and Dgat2 between the MI/R and Control groups by western blotting. The results showed that the protein expression levels of Dgat2 was consistent with that of mRNA (P<0.05) (**Figures 5I, J**). Further Immunohistochemical staining confirmed that myocardial Dgat2 expression in the MI/R group was significantly decreased compared to the control group (**Figures 5K, L**). Subsequent animal experiments revealed sustained DGAT2 downregulation at 24 hours post-ischemia/reperfusion. Notably, analysis of an independent dataset (GSE61592) confirmed this differential expression pattern persisted through 72 hours (**Supplementary Figure 1**), corroborating our experimental observations.

**Table 1:** Primer sequences for mRNAs.

Gene name	5’-3’ sequence	
	Forward	Reverse
Gapdh	AGGTCGGTGTGAACG GATTTG	TGTAGACCATGTAGTTG AGGTCA
Cybb	TGTGGTTGGGGCTG AATGTC	CTGAGAAAGGAGAGCAG ATTTCG
Dgat2	GCGCTACTTCCGAG ACTACTT	GGGCCTTATGCCAG GAAACT

### 3.6 PPI analysis and functional enrichment of the hub MitoDEG Dgat2

**Figure 6:**
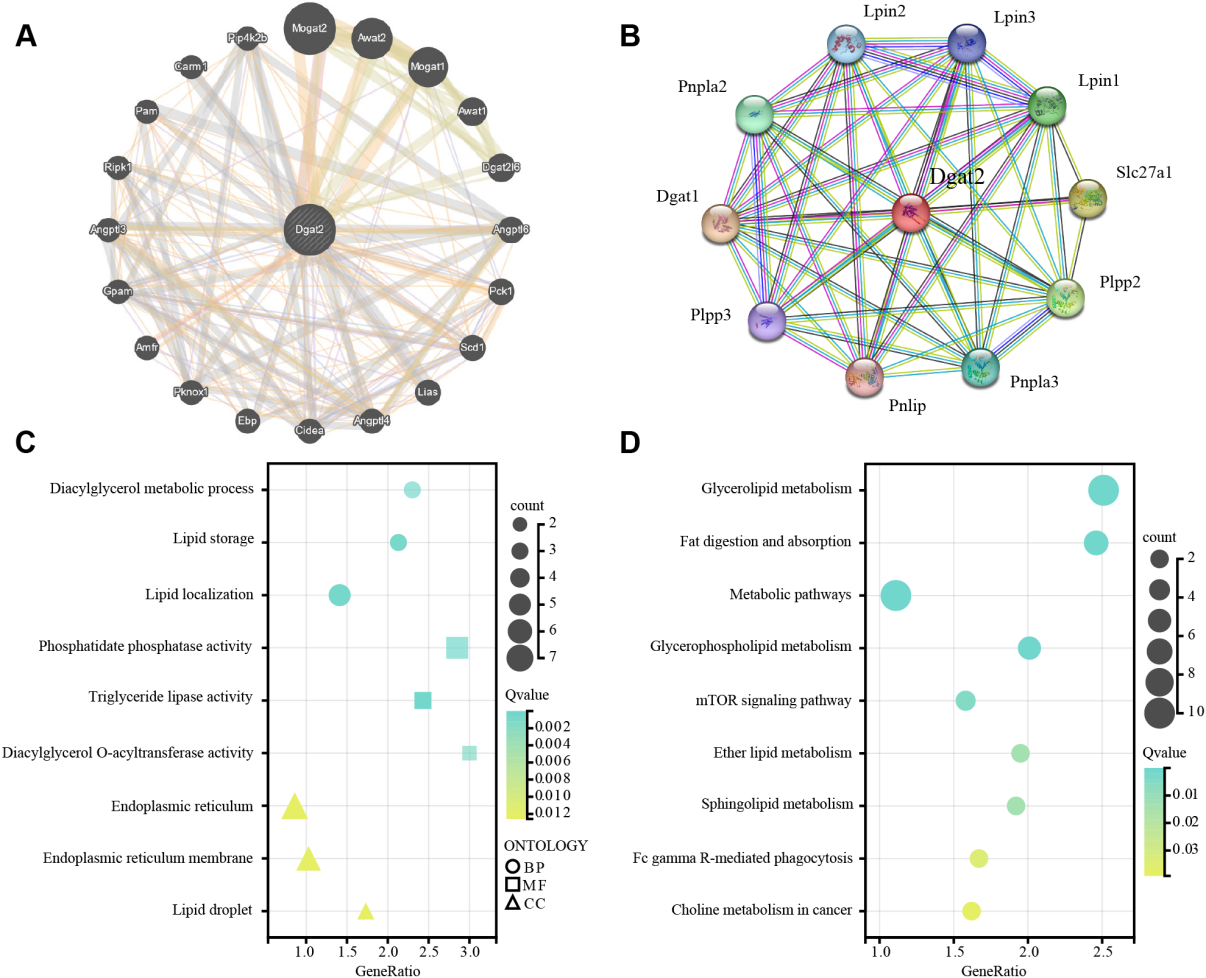
PPI network analysis, GO and KEGG enrichment analyses of Dgat2. **(A)** PPI Network Analysis for Dgat2 of GeneMANIA Database. The edge colors represent different types of interactions: physical interactions (red, 45.00%), predictions (yellow, 22.45%), co-expression (green, 17.96%), pathways (light blue, 2.07%), genetic interactions (green, 1.81%), co-localization (dark blue, 1.57%), shared protein domains (yellow, 1.04%), and other predicted associations (gray, 8.09%). **(B)** PPI Network Analysis for Dgat2 of STRING Database. Based on the confidence scores (ranging from 0 to 1) provided by the STRING database, we selected the top 10 highest-confidence protein-protein interactions with a confidence score ≥ 0.9 for visualization. **(C)** The enriched GO terms for Dgat2(BP biological process, CC cellular component, MF molecular function). GeneRatio represents the proportion of enriched genes in each term; the color of the dots indicates the number of genes (count), and the size of the dots reflects the level of significance (Q-value). **(D)** KEGG pathway enrichment results for Dgat2.

The interaction of Dgat2 with other proteins visualized by GeneMANIA was shown in **Figure 6A**. As shown in **Figure 6B**, we explored potential molecular interactions of Dgat2 using PPI network analysis of the STRING database. For verification purposes, detailed interaction data—including interacting protein pairs, STRING confidence scores, and supporting evidence types—are provided in **Supplementary Table 1**.

The biological processes, cellular components, and molecular functions analyzed in GO enrichment were dominated by lipid metabolism processes as well as the important molecules and their functions therein, respectively (**Figure 6C**). Based on the KEGG database to further decipher the underlying biological pathways, it was found that the enriched molecular pathways included Glycerolipid metabolism, Fat digestion and absorption, Metabolic pathways, Glycerophospholipid metabolism, mTOR signaling pathway, Ether lipid metabolism, Sphingolipid metabolism, Fc gamma R-mediated phagocytosis, Choline metabolism in cancer (**Figure 6D**). These findings are in general agreement with the results of GO enrichment analysis, which affirmed that Dgat2 has a role in biological oxidation and lipid metabolism.

### 3.7 Immune infiltration analysis of Dgat2

**Figure 7:**
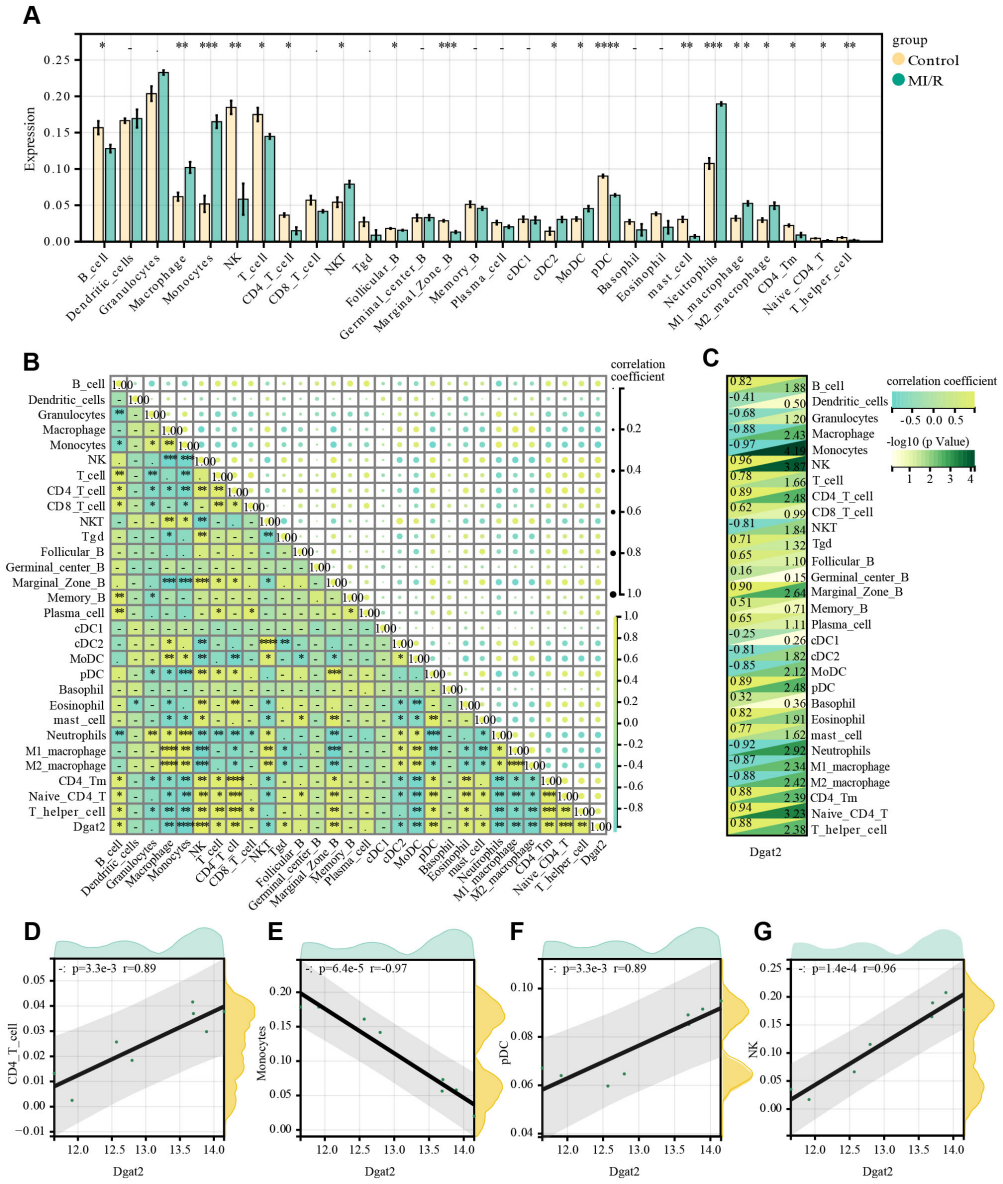
Infiltration of immune cell types compared between MI/R and Control group. **(A)** The histogram of the immune cell proportions; **(B)** The correlation matrix of immune cell proportions; **(C)** Heatmap of immune cells correlating with Dgat2; **(D-G)** Four leukocyte subpopulations(CD4^+^ T cell, Monocytes, pDC and NK) with significant correlation with the expression of the key gene Dgat2.

ImmuCellAI calculated the proportions of 24 leukocyte subpopulations in sham-operated mice and mice treated with I/R for 24 h, respectively, and whether there was a correlation between changes in leukocyte subpopulations. Analysis of the immune microenvironment in mice treated with I/R for 24 h versus mice subjected to sham surgery showed significant differences in the abundance of 19 immune cell types. These differences were statistically significant (**Figures 7A, B**). This suggests that the ischemia-reperfusion process in cardiomyocytes has varying degrees of infiltration of multiple immune cells in mice, and that these immune cell infiltrations may be potential points of modulation for treatment.

We also explored the relationship between key genes and immune infiltrating cells during ischemia-reperfusion treatment or not, and found a correlation between the expression of Dgat2 and that of certain leukocyte subpopulations (**Figure 7C**), for example, CD4_T_cells, Monocytes, pDC, NK (**Figures 7D–G**). This suggests that Dgat2 has a role in modulating the immune microenvironment during myocardial ischemia-reperfusion.

### 3.8 Transcription factor prediction

**Figure 8:**
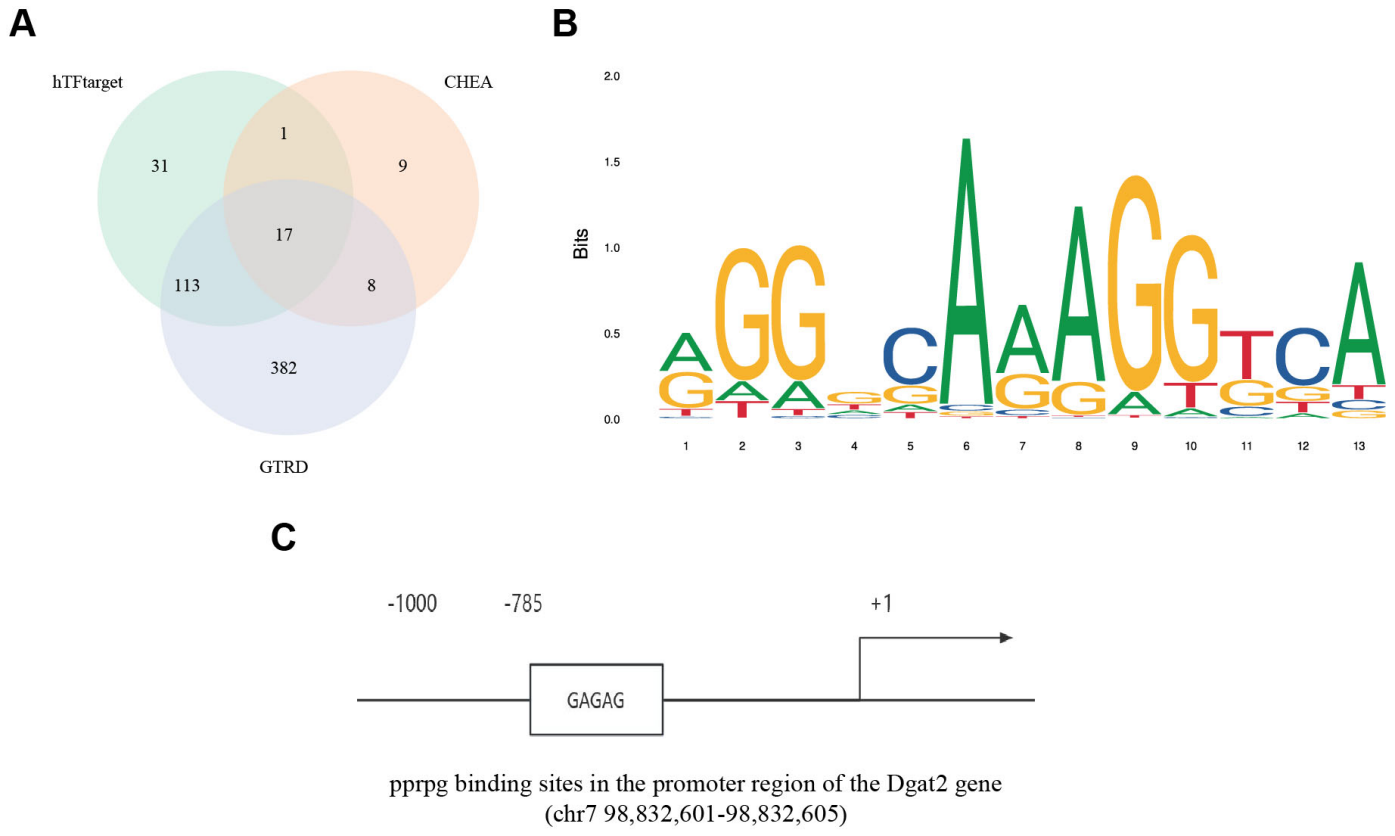
Transcription factor prediction for Dgat2. **(A)** Three different databases for GTRD, ChEA3, and hTFtarget identified 17 transcription factor overlaps; **(B)** PPARG binding site prediction by JASPAR; **(C)** Conserved PPARG binding sites in the promoter region of the DGAT2 gene.

To explore the reasons for the low expression of Dgat2 in MI/RI mice, we analyzed the Dgat2 transcription factor binding sites in the promoter region of the gene (transcription start site -2000 bp~500 bp). Multiple prediction methods identified 17 overlapping transcription factors (**Figure 8A**), and in combination with previous pathway enrichment and PPI, we noted the transcription factor PPARG.

The UCSC results showed the presence of a conserved sequence in the promoter region of the Dgat2 gene as a PPARG binding site with high confidence (**Figure 8C**). This is consistent with the finding that the function of the Dgat2-encoded protein is tightly correlated with PPARG in the PPI results and suggests that functional down-regulation of the PPARG-related pathway may play an important role in causing down-regulation of Dgat2 expression.

Subsequently, we performed DNA binding site prediction of PPARG protein, and the figure shows the baseline of the functional region of PPARG as a transcriptional regulator (**Figure 8B**). The analysis showed that the binding sequences of the DNA with the highest confidence of PPARG protein and the sequences of the high-confidence PPARG protein binding site in the promoter region of Dgat2 gene were basically the same. This further confirms that PPARG plays an important role in the transcriptional regulation of Dgat2.

## 4 Discussion

In acute ischemic heart disease (for example, acute myocardial infarction), following quick therapy restores the volume of the ischemic myocardium, the injury worsens temporarily, resulting in myocardial ischemia/reperfusion injury. Myocardial ischemia/reperfusion is a complex process involving metabolic and immune factors (30), which is common in cardiovascular diseases. Its mechanism is complex and not fully understood, involving multiple biological processes such as oxidative stress, calcium imbalance, mitochondrial damage, energy metabolism disorders, apoptosis, and inflammatory responses (31). These biological processes interact to generate changes in the internal and exterior milieu of cardiomyocytes, thus aggravating cardiac damage, and no effective treatment has been established (30). As a result, screening for new molecular markers is important to understanding illness onset and progression.

In this study, differentially expressed genes (DEGs) associated with myocardial ischemia/reperfusion were identified through differential analysis of GSE160516 datasets. These DEGs were found to be enriched in mitochondrial function, immune inflammation, neutrophil extracellular trap (NET) formation, and respiratory and oxidative stress-related pathways. Previous research has shown that myocardial ischemia/reperfusion activates an inflammatory response, leading to expansion of myocardial infarction and myocardial remodeling, while multiple mechanisms, including altered metabolic status, work together to modulate the activation and function of immune cells in response to MI/RI (32). Ischemia/reperfusion is strongly associated with respiratory and oxidative stress (14) and NETs are present in the damaged myocardium (14, 33). Our findings are consistent with previous literature publications. We next identified mitochondria-associated differential genes (MitoDEGs) and revealed that these 65 MitoDEGs were enriched in pathways related to mitochondrial function, oxidative stress, and fatty acid metabolism. Finally, we identified two key genes, Dgat2 and Cybb, using machine learning and PPI network analysis. Furthermore, we constructed a mouse model of ischemia/reperfusion (I/R) for 24 h, and using RT-PCR and Western Blot, we discovered that only Dgat2 was significantly under-expressed in the I/R 24 h model at the same time, which was consistent with the bioinformatics results. Therefore, we will focus on Dgat2 in the following research.

Diacylglycerol O-acyltransferase 2 (Dgat2) is a gene necessary for triglyceride synthesis and cell membrane architecture. It encodes acetyl-CoA (Acetyl-CoA) (34), which is required for lipid anabolism and regulation (35). In our functional enrichment analysis, Dgat2 was strongly enriched in the mTOR signaling pathway, which is known to prevent myocardial damage by suppressing autophagy in myocardial ischemia-reperfusion (36). This suggests that Dgat2 may play a regulatory role in MI/RI. Recent investigations have demonstrated that, although Dgat2 is primarily active in the endoplasmic reticulum, it is also directly associated with mitochondrial function, regulating lipid metabolism and energy balance within mitochondria (37, 38), and has a significant impact on mitochondrial structure and function (39, 40). Mitochondria, the primary energy-producing centers in cells, produce acetyl-CoA (Acetyl-CoA) through β oxidation of fatty acids. This molecule is directly implicated in the Dgat2-mediated triglyceride synthesis pathway (41). This direct relationship links mitochondrial energy production to lipid synthesis, which influences cellular energy metabolism and fat storage. Phospholipids and triglycerides found on mitochondrial membranes help to maintain cellular energy balance and antioxidant capability by regulating mitochondrial membrane potential and ATP-producing efficiency (42). These findings further suggest that Dgat2, a mitochondria-related gene, is closely related to the regulation of cells’ ability to cope with oxidative stress (43) and may play a role in myocardial ischemia-reperfusion processes. All malignancies (35), nonalcoholic fatty liver disease (NAFLD), and type 2 diabetes (44) have been linked to abnormal Dgat2 expression. This aberrant expression is mostly seen in three areas: oxidative stress, lipid metabolism, and cell death. However, investigations have only found that Dgat2 may have some heart-protective properties (45), and its significance in myocardial ischemia/reperfusion has not been published.

Myocardial ischemia/reperfusion injury is predominantly caused by oxidative stress-induced mitochondrial dysfunction (46), which further leads to apoptosis (programmed cell death) and necrosis of cardiomyocytes (47). Numerous studies have shown that disorders of lipid metabolism and oxidative stress are key factors in cardiomyocyte death (48–51). Dgat2, as a key gene regulating lipid metabolism, mitigates myocardial ischemia-reperfusion injury by maintaining mitochondrial membrane lipid homeostasis (phospholipid/triglyceride) and energy metabolism (β oxidation-acetyl-CoA) functions, protecting mitochondrial integrity and enhancing cellular antioxidant capacity (52, 53). During myocardial ischemia, coronary artery occlusion or stenosis exacerbates myocardial ischemia (45, 54), leading to oxidative stress, cell membrane damage, mitochondrial dysfunction, and apoptosis (55). In addition, intracellular reactive oxygen species (ROS) are primarily derived from the cytoplasm and mitochondria (14) and these ROS cause mitochondrial damage through different pathways, which play an important role in the etiology of many myocardial ischemia/reperfusion injuries (56–58). It has also been found that the expression level of Dgat2 in cardiac tissue changes during myocardial ischemia/reperfusion injury (59, 60). Dgat2 may protect cardiomyocytes during ischemia/reperfusion by regulating triglyceride synthesis and other lipid-related protective mechanisms by influencing cardiomyocytes’ resistance to oxidative stress. Our bioinformatics analysis further revealed that Dgat2 expression was significantly reduced in ischemia/reperfusion injured cardiomyocytes. Therefore, we hypothesized that modulating the expression or activity of Dgat2 may affect the sensitivity of cardiomyocytes to ischemia/reperfusion injury, which in turn alters the severity and recovery process of myocardial injury. This finding provides an important basis for further research on the protective effect of Dgat2 in myocardial ischemia/reperfusion injury and its potential clinical application value.

Furthermore, we predicted transcription factors (TFs) upstream of Dgat2 using the JASPAR database and the TF target prediction website, revealing an overlapped important TF, PPARG. PPARG has a protective effect on the heart, and its activation can inhibit the inflammatory response, alleviate oxidative stress, and promote angiogenesis, so lowering the severity of MI/RI and maintaining cardiac function (61). Thiazolidinediones, as ligands for PPARG, have been shown to reduce tissue necrosis associated with acute myocardial infarction (62). We found multiple PPARG binding sites in the promoter region of the Dgat2 gene. Considering that the protein encoded by Dgat2 and PPARG function in the same pathway (34, 63), we discovered that the prediction of the DNA binding site of PPARG was basically consistent with the conserved sequence of the Dgat2 promoter region. In previous studies on hepatic glucose and lipid metabolism, drug treatment led to an upregulation of PPARG expression, which was associated with a downregulation of Dgat2 expression (63). However, there has been no confirmation in myocardial or ischemia-reperfusion models. Therefore, we speculate that PPARG may be an essential target for regulating Dgat2 expression, and this potential regulatory pathway may provide a novel therapeutic strategy for myocardial ischemia-reperfusion injury, which is worthy of further investigation.

It has been reported that metabolic status and immunological processes are tightly connected with ischemia/reperfusion (64). This aligns with our observations. Leukocytes are first recruited to the area of ischemia and infarction after being stimulated by local and systemic signals associated with cell damage and necrosis (65). Our investigation found a link between Dgat2 expression and the expression of specific immune cells, including alterations in neutrophils (66) monocytes (67) and T lymphocyte subsets (32), which were also key factors in the pathogenesis of myocardial ischemia/reperfusion. Numerous studies have shown that mitochondrial dysfunction during MI/RI triggers a DAMPs gradient (mtDNA/ATP/HMGB1) that directly activates these immune subsets (68). CD4+ T cells are driven by mtDNA-TLR9 signaling in dendritic cells, which promotes IL-12 secretion and Th1/Th17 polarization, while the CD36-TCR axis further amplifies IFN-γ production to exacerbate cardiomyocyte necrosis (69). Concurrently, NK cells exhibit enhanced perforin/granzyme B release via mtDNA-HMGB1-NKG2D interaction (70, 71). Neutrophils undergo caspase-11-mediated NETosis driven by mitochondrial ROS overproduction, which is synergistically amplified by mtDNA-TLR9 crosstalk (72). In parallel, monocytes activate NLRP3 inflammasomes through HMGB1-RAGE binding, an effect counteracted by DGAT2-mediated lipid raft stabilization (70, 73). These mechanisms establish a feedforward loop where mitochondrial damage fuels immune activation, reciprocally exacerbating mitochondrial injury via ROS/NLRP3/NETs pathways.

In this study, Dgat2, a critical gene for the interaction between mitochondrial metabolism and myocardial ischemia/reperfusion was discovered for the first time through bioinformatics analysis. Subsequently, we performed further experimental validation to highlight the potential role of downregulation of this gene expression in myocardial ischemia/reperfusion. However, we acknowledge that the study still has some limitations. First, we validated the correlation of Dgat2 with mitochondria in ischemia/reperfusion cardiomyocytes in *in vitro* and *in vivo* models, but the regulatory mechanisms and hierarchical networks associated with Dgat2 and mitochondria and MI/RI are not fully understood, and further knockdown or overexpression studies to confirm the regulation of DGAT2 by PPARG are needed. Second, changes in Dgat2 expression and its immunological changes associated with different reoxygenation or reperfusion times should also be comprehensively assessed. Third, although the animal models we used can mimic the morphological features of human MI/RI, they cannot encompass the natural history and histological features, which could lead to potentially erroneous or inconsistent conclusions, thus limiting the generalizability of the study. Therefore, in clinical practice, we need to conduct additional clinical cohort studies and validation.

## 5 Conclusion

In conclusion, we discovered Dgat2, a new potential mitochondria-related gene target in myocardial ischemia/reperfusion by comprehensive bioinformatics analysis and molecular experiments. This finding is valuable for clinical genetic screening and provides important clues for the development of new therapeutic strategies.

## Data Availability

The original contributions presented in the study are included in the article/**Supplementary Material**. Further inquiries can be directed to the corresponding author.
