# Supplementary figures and images for: Comprehensive bioinformatics analysis and experimental verification identify mitochondrial gene Dgat2 as a novel therapeutic biomarker for myocardial ischemia-reperfusion
Source: Front Endocrinol (Lausanne). 2025 May 29;16:1539646. doi: 10.3389/fendo.2025.1539646 (PMC12159077; doi:10.3389/fendo.2025.1539646)

Uncut Western Blot Gel

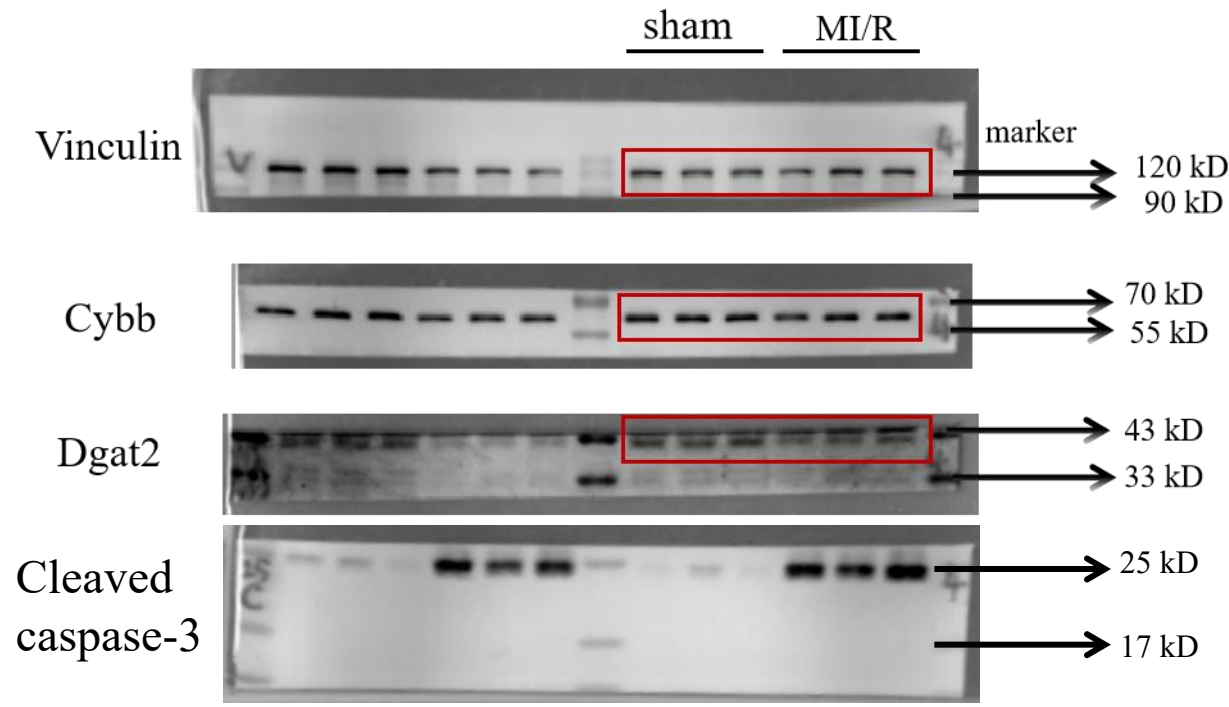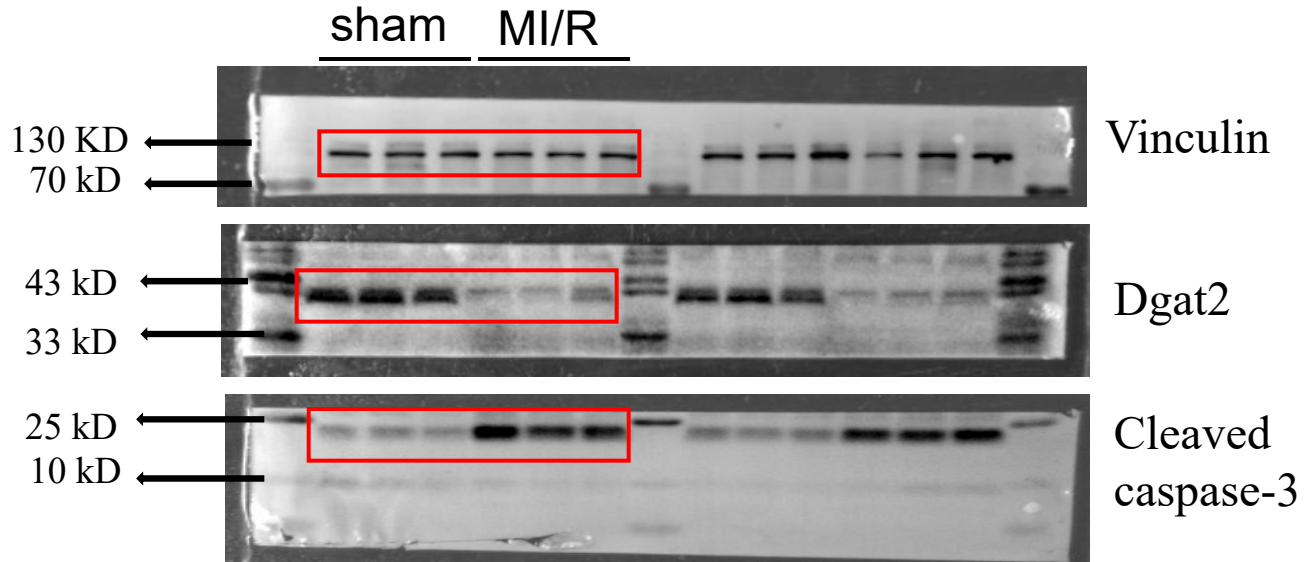

# Uncut Western Blot Gel

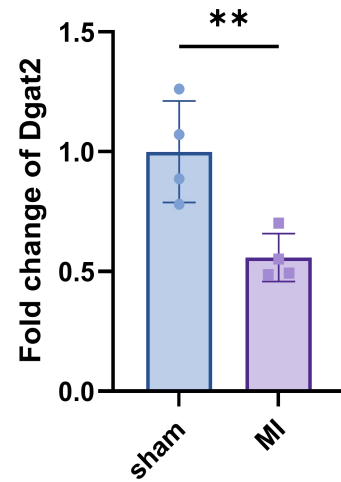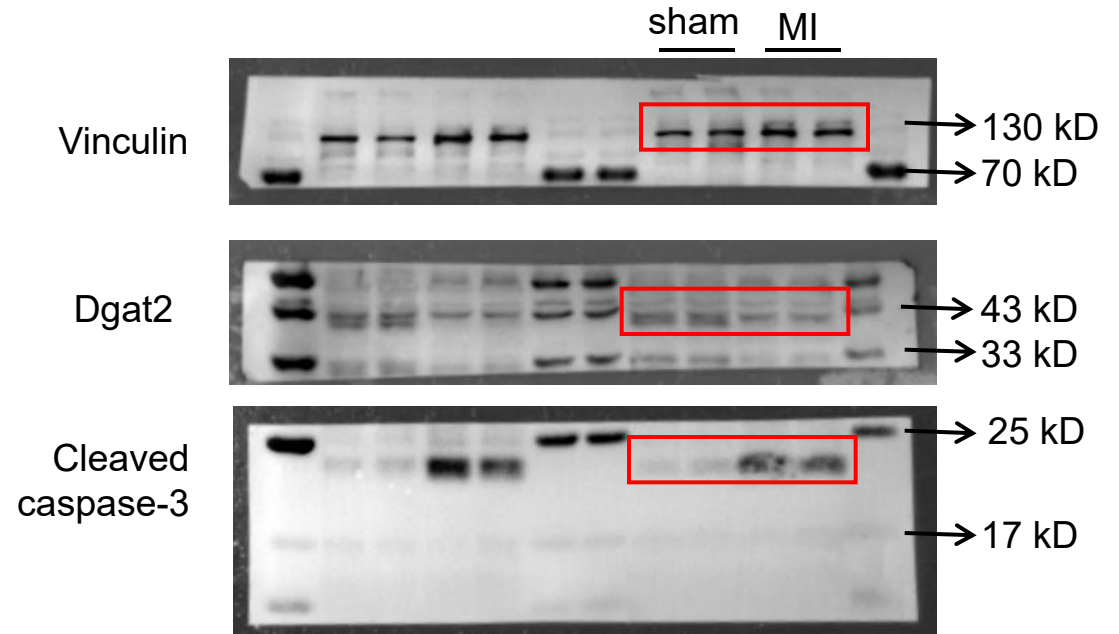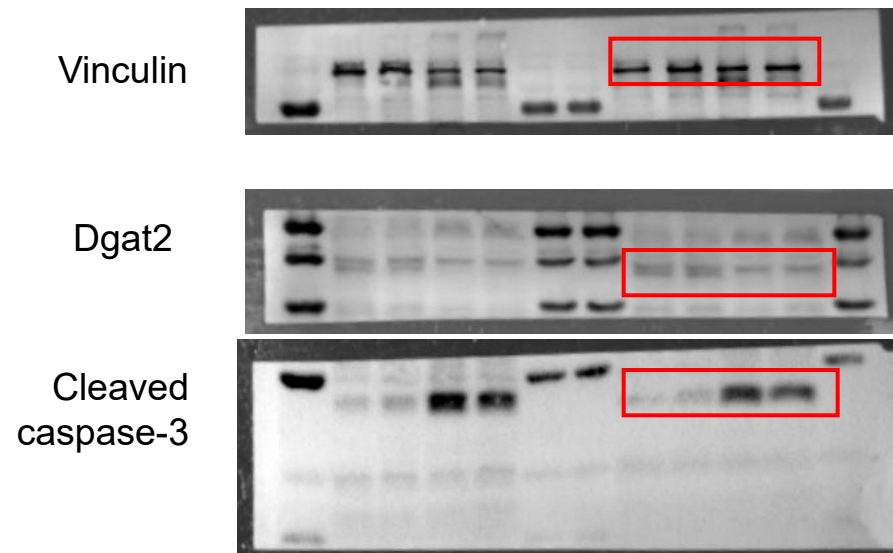

Supplement: Supplementary file 1 [file DataSheet1.pdf]

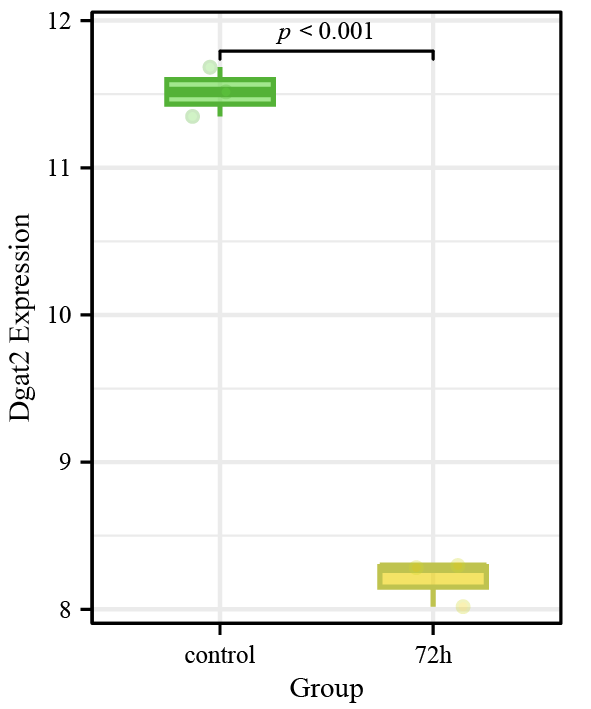

Supplement: Supplementary file 3 [file Image1.tif]
