# Supplementary material for: Comprehensive bioinformatics analysis and experimental verification identify mitochondrial gene Dgat2 as a novel therapeutic biomarker for myocardial ischemia-reperfusion
Source: Front Endocrinol (Lausanne). 2025 May 29;16:1539646. doi: 10.3389/fendo.2025.1539646 (PMC12159077; doi:10.3389/fendo.2025.1539646)
Supplement: Supplementary file 4 [file Table1.docx]

**Supplementary Table 1.** Detailed protein-protein interaction data underlying Figure 6B.

| node1 | node2 | score | Evidence Types |
| --- | --- | --- | --- |
| Dgat2 | Dgat1 | 0.995 | Co-Expression, Co-Mentioned in Pubmed Abstracts,  Association in Curated Databases, Experimental/Biochemical Data |
| Dgat2 | Lpin1 | 0.97 | Co-Expression, Co-Mentioned in Pubmed Abstracts,  Association in Curated Databases, Experimental/Biochemical Data |
| Dgat2 | Lpin2 | 0.956 | Co-Mentioned in Pubmed Abstracts,  Association in Curated Databases, Experimental/Biochemical Data |
| Dgat2 | Lpin3 | 0.946 | Co-Expression, Co-Mentioned in Pubmed Abstracts,  Association in Curated Databases, Experimental/Biochemical Data |
| Dgat2 | Plpp2 | 0.963 | Co-Mentioned in Pubmed Abstracts,  Association in Curated Databases |
| Dgat2 | Plpp3 | 0.941 | Co-Expression, Co-Mentioned in Pubmed Abstracts,  Association in Curated Databases |
| Dgat2 | Pnlip | 0.94 | Co-Mentioned in Pubmed Abstracts,  Association in Curated Databases |
| Dgat2 | Pnpla2 | 0.978 | Co-Expression, Co-Mentioned in Pubmed Abstracts,  Association in Curated Databases |
| Dgat2 | Pnpla3 | 0.962 | Co-Expression, Co-Mentioned in Pubmed Abstracts,  Association in Curated Databases |
| Dgat2 | Slc27a1 | 0.986 | Co-Expression,Co-Mentioned in Pubmed Abstracts,  Experimental/Biochemical Data |
